# Implementing Perennial Kitchen Garden Model to Improve Diet Diversity in Melghat, India

**DOI:** 10.5539/gjhs.v8n4p10

**Published:** 2015-07-30

**Authors:** Tannaz J. Birdi, Shimoni U. Shah

**Affiliations:** 1 The Foundation for Medical Research, Worli, Mumbai, India

**Keywords:** diet diversity, green leafy vegetables, kitchen garden, Melghat

## Abstract

Lack of diet diversity causing micronutrient deficiency is common in developing countries and is gaining attention due to the hidden consequences of impaired physical and cognitive development. This paper describes the propagation of a sustainable perennial kitchen garden (KG) model to address household (HH) diet diversity in Melghat. Nutrient dense plants, comprising of minimum one tree (perennial) and one green leafy vegetable (GLV) were given to participating HHs along with qualitative interventions. Baseline survey was conducted in winter 2011 followed by seasonal surveys over 2 years to record changes in KG practices, dietary intake and childcare practices. Marked increase from 4% at baseline to 95% at endline was seen in the KG maintainance. Increased diversity was seen in all food categories other than cereals and pulses. Variety of GLVs consumed increased over the two winters as well as the 2 summers. However, no change in the quantity of GLV consumed was noted which was attributed to the duration of the study period being insufficient for the trees to grow and provide adequate leaves for consumption. Notably, livelihood component was not promoted and HHs were encouraged to harvest and distribute excess seeds to relatives and neighbours. The study generated huge demand from HHs within the intervention and neighbouring villages. It concludes that a well designed perennial KG along with imparting adequate knowledge can be a sustainable practice to increase diet diversity and GLV intake which would help address micronutrient deficiencies in the community.

## 1. Introduction

Micronutrient deficiency in developing countries is common and has gained increasing attention (WHO, 2007) due to its contribution to hidden consequences like impaired physical and cognitive development and work productivity (Demment, Young, & Sensenig, 2003). A nation-wide survey in India indicates diets deficient in micronutrients (Brahmam, 2007). Therefore, overcoming this requires effective and efficient strategies that use available resources and have long term benefits.

Fortification and supplementation are some measures which are popularly undertaken in most countries to correct micronutrient deficiencies. These strategies may not be accessible, acceptable or sustainable in poor rural communities where the burden of nutritional deficiency is greatest (Underwood, 2000; Tontisirin, Nantel, & Bhattacharjee, 2002; Imhoff-Kunsch, Flores, Dary, & Martorell, 2007). Supplementation is often inconsistent since it is reliant on funding (Fiedler, 2000). In addition, experiences with vitamin A and iron supplementation in India have reported improper supply chains, co-ordination between health staff, dependence on health care system for delivery and inadequate compliance (Vijayaraghavan, 2002; Bamji, 2011). In case of fortification, purchasing power and distribution systems pose limitations although the cost-benefit analysis is better. In India, iron fortified wheat flour, oil with vitamin A and D and micronutrient sprinklers have also been tried (Bamji, 2011). Both strategies are technology driven and monitoring of quality is important (Mannar & Snakar, 2004). Research gaps remain with respect to doses, absorption and effects of fortification (WHO, 2010; Bamji, 2011). Therefore feasibility, acceptance and impact in India still need to be studied (Bamji, 2011). Strategies such as use of food vehicles devised in other countries may not be suitable in India.

Sustainability of any nutritional intervention is paramount (Nantel & Tontisirin, 2002) and it is this component of sustainability that remains unaddressed by these solutions ((Demment, Young, & Sensenig, 2003; Ramakrishnan & Darton, 2002) and should not be considered as substitutes to a well balanced diet (Allen, Benoist, Dary, & Hurrell, 2006; Flynn et al., 2009).

Reviews of evidence from Africa and Asia suggest that interventions targeting home gardening and household animal produce have the potential to contribute to improved household nutrition (Ruel & Levin, 2000; Leroy & Frongillo, 2007; Hawkes & Ruel, 2010; Berti, Krasevec, & FitzGerald, 2004). Practice of kitchen gardens (KG) is a long standing tradition in India. They are shown to significantly improve diet diversity, strengthen interaction within communities, improve health as well as significantly reduce food insecurity which is brought about by worrying about money required for food (Carney et al., 2012). Whilst they have been the focus of development activities for over a century, the purpose &/or scope of the gardens promoted by development agencies has changed. Recently the emphasis has been on KGs for addressing single micronutrient deficiencies, mainly vitamin A (Girard, Self, McAuliffe, & Oludea, 2012). Since communities usually suffer from more than one micronutrient deficiency it is advisable to look at this approach more holistically.

Most food based interventions like KG tend to fail as their main focus is on agriculture and nutrition. Focusing on socio-economic factors plays a key role in ensuring sustainability of these projects. Assuming that increased production of diversified foods will lead to increased consumption and improved intake of micronutrients is one of the limitations that need to be tackled. Understanding and adapting to cultural beliefs, social norms and local food habits will ensure viability of these interventions (Burchi, Fanzo, & Frison, 2011).

This paper describes the propagation of a perennial KG model along with education to try and address the issue of micronutrient deficiencies in Melghat by increasing the household diet diversity. This region in Amravati district of Maharashtra faces extreme adverse climatic conditions throughout the year accompanied by acute water shortage that affects cultivation. It also has amongst the highest numbers of malnutrition and infant mortality cases (Talapalliwar & Garg, 2014; Malnutrition Monitoring Committee, 2007-12). In an earlier publication (Birdi, Joshi, Kotian, & Shah, 2014) the possible reasons for the high malnutrition levels in this area and the factors affecting good nutrition have been documented and discussed.

The present perennial KG model is an attempt to address sustainability which is seen to be one of the major problems of KG once the motivating NGO withdraws. Results of participant households before and after the intervention are reported and compared to observe the impact of the perennial KG on their diet.

## 2. Method

### 2.1 Study Design

Ten villages that were geographically representative of the Dharni block were selected for the KG intervention. List of households (HHs) that had at least one child aged <6 years in these villages was obtained from the *Anganwadis* (AWs) of which 396 HHs were selected based on voluntary participation. A signed written consent was obtained from an adult member of the HH that was interviewed at baseline. Baseline findings (Birdi, Joshi, Kotian, & Shah, 2014) were collected in winter 2011. KG plant distribution and qualitative interventions were provided to these HHs which were subsequently followed for 2 years to record seasonal changes in KG practices, dietary intake and childcare practices. Since many families migrated, follow up of 203 HHs during summer 2012, 198 HHs during monsoon 2012 and 259 HHs during winter of 2012 could be undertaken. End-line was carried out in summer 2013 with 362 HHs.

### 2.2 KG Plant Distribution

A total of 18 plants that were nutrient dense and/or hardy, comprising of a core group of perennials, fruit trees, vegetables and green leafy vegetables (GLVs) were distributed to HHs. Minimum of one tree (perennial) and one GLV were given to all HHs and additional plants were distributed based on demand from the community as well as survival of the plants initially distributed.

Trees and climbers were distributed in the monsoon. First year monsoon distribution was that of papaya, drumstick, tamarind and *agathi* which were also distributed in the second year along with winged bean, finger millet, custard apple, Indian red spinach and field bean. Banana saplings and GLV's like *chowlai*, *chakavat*, *ambat chuka* and garden cress were distributed in winter 2011 and 2012. Vegetables like cauliflower and turnip were distributed during summer 2012 along with curry leaf saplings. Groundnut was distributed during both summers.

### 2.3 Qualitative Interventions

Community interactions and home visits continually emphasized nutrition and other inter-related components through IEC activities. Importance of nutrition and role of KG was explained along with importance of incorporating the intervention plants in the diet. Childcare practices with a stress on complementary feeding which could include some of the intervention plants like papaya and banana were also carried out.

### 2.4 Quantitative Data Collection

Any one adult member (>21 years) that was available in the HH was interviewed at baseline, three follow-ups and end-line. At baseline, data on demographics, land holding, income sources, migration, existing KG practices, PDS, AW, child care practices, and immunization services, water hygiene and sanitation was collected by means of a structured questionnaire along with dietary intake patterns and food provisioning habits of HHs. At every follow-up, data on signs and symptoms of micronutrient deficiencies, KG practices and dietary intake patterns and food provisioning habits of HHs were evaluated to obtain any change in diet diversity and quantity of food intake. End-line was structured as baseline for comparison purpose.

### 2.5 Addition of New HHs

New HHs from baseline villages and 5 additional villages showed interest in joining the study during the intervention phase. These HHs were given intervention plants based on demand and availability. For comparison purpose, 411 demand generated HHs were surveyed to obtain their reasons for participation and having a KG, existing KG practices and food habits before and after the intervention plants were given.

### 2.6 Data Analysis

All the quantitative data collected during baseline, follow-ups and end-line were entered in CSPro 4.1 and imported into SPSS v19 for analysis. Daily diet, weekly provisioning of food items and KG practices were seasonally compared before and after intervention.

### 2.7 Ethical Consideration

Clearance was obtained from the Institutions’ Ethics Committee (Institute/IEC/KG/01/2012).

## 3. Results

### 3.1 Baseline Assessment

#### 3.1.1 Prevalence of Malnutrition and Micronutrient Deficiency

As per our earlier findings (Birdi, Joshi, Kotian, & Shah, 2014) more than half the children (1-10 years) and adolescents (11-19 yrs) were severe to moderately underweight. Amongst the other age groups, 18% of adults in the reproductive age group (20-45 years) and 25% of members >45 years were also found to be severe to moderately underweight.

Assessment of anaemia and vitamin B deficiency was estimated during all three seasons in 2012 and an average estimation of the burden of micronutrient deficiency was obtained amongst adults and children. Data revealed Vitamin B deficiency viz. glossitis and stomatitis in 20% adults and 15% of children with no apparent gender bias. Anaemia was diagnosed by observing eyes, tongue and nails. It was estimated that 55% adult women and 25% girls were anaemic whereas only 35% adult men and 20% boys showed signs of anaemia. This data indicated that the region not only suffers from protein calorie malnutrition but also from micronutrient deficiency.

#### 3.1.2 Cropping Pattern in Fields

Approximately 50% of the families (154 HHs) with complete irrigation facilities cultivated crops in all three seasons whereas those with no irrigation facility cultivated crops in only one season (Table 1). GLVs and other vegetables were often planted in the field along with *rabi* crops as opposed to their backyard.

**Table 1:** Correlation of irrigation with cropping pattern

Irrigation Facility	% Households		

	one season	two seasons	three seasons
No irrigation	60	40	1
Fully irrigated	0	47	53
Partially irrigated	8	78	14

Almost all HHs cultivated crops during monsoon (99% HHs) followed by 66% HHs during winter and only 14% HHs cultivated crops during summer. Pulses and cereals were most commonly cultivated in the field along with cash crops like soya bean and cotton (Table 2).

**Table 2:** Field crops most frequently cultivated in various seasons - % HHs

Monsoon	Winter	Summer
*Tur* (Cajanus cajan) – 80	Bengal gram – 53	Green gram – 13
*Jowar* (Sorghum vulgare) – 77	Wheat – 46	
Soya bean – 75	*Masoor* (Lens esculenta) – 12	
Rice – 71		
Cotton – 39		
*Chavli* (Vigna catjang) – 13		

#### 3.1.3 Traditional KG

Baseline findings revealed that KG's were already being practiced by 79% of the HHs. Common reasons cited for maintaining a KG were that vegetables were more expensive to buy from outside (80%) and convenience (30%). HHs did not practice KG (21%) mainly due to unavailability of land (62%) or water (33%) and also due to requirement of additional effort (14%). Flowering plants however, were often planted despite water shortage hinting that growing vegetables may not be a priority. The area available for KG varied widely and was often not used entirely for cultivation. Forty three percent HHs had upto 50 m^2^ of KG land, 24% had 51 m^2^ – 100 m^2^ and 33% HHs had > 100 m^2^ of KG land. Backyard was also often used to rear livestock. Some commonly cultivated plants in the KG are listed in Table 3. Seeds for the KG plants were mainly indigenous (77%), bought from the market (58%) or obtained from relatives or neighbours (27%).

**Table 3:** KG Plants commonly cultivated in various seasons (%HH)

Monsoon – 98% HH	Winter – 33% HH	Summer – 4% HH
Pumpkin (64)	Fenugreek (24)	Fenugreek (3)
Field bean (12)	Spinach (20)	Spinach (2)
Brinjal (12)	Coriander (19)	Brinjal (2)
	Radish (12)	Coriander (1)
	Brinjal (11)	

### 3.2 Introduction of a Perennial KG

Besides the limited availability of space in the backyard, it was also noted that only 4% of HHs cultivated vegetables in their KG during summer (Birdi, Joshi, Kotian, & Shah, 2014). Summer was when availability and affordability of food items was the least and therefore, an attempt was made to implement a perennial KG model by diversifying the already existent traditional KGs along with imparting nutrition education.

The plants considered for distribution were perennial, micronutrient dense, required minimal maintenance and could survive in harsh climatic conditions. Hybrid seeds/saplings were not distributed in order to promote self sufficiency. Since long term sufficiency and sustainability were priorities, trees and climbers with edible leaves and other parts were preferred. Although trees require time to mature, they are more beneficial in the long run compared to GLVs which have a short cultivation cycle but require constant replanting. Unlike GLVs, consumption of leaves from trees and climbers is possible round the year. They provided higher yield per unit area making them suitable for HHs with smaller plots. Trees also require less water compared to GLVs and could be therefore grown around the household washing area on waste water since most HHs used soil and ash for washing utensils. While the trees reached maturity, an interim supply of seeds of GLVs were distributed to sustain motivation.

In the course of the project, based on the above criteria, 18 plants were distributed (Table 4). Many of these plants were new or not widely available in the region. It was these plants that the HHs were more enthusiastic about during community interactions.

**Table 4:** List of plants distributed for planting in KGs

Intervention Plants	Type	Material distributed (HH%)	Planted (HH%)
Drumstick (*Moringa oleifera* Lam.)	Tree (p)	seed, sapling (79)	100
Papaya (*Carica papaya* L.)	Tree	sapling (84)	100
Banana (*Musa paradisiaca* L.)	Tree	sapling (56)	100
Garden Cress (*Lepidium sativum* L.)	GLV	seed (75)	86
Indian red spinach (*Basella rubra/alba* L.)	Climber (p)	seed, cutting (80)	100
Cauliflower (*Brassica oleracea* L.)	Vegetable	seed (5)	100
Turnip (*Brassica rapa* L.)	Vegetable	seed (16)	72
*Agathi* (*Sesbania grandiflora* L.)	Tree (p)	seed, sapling (45)	100
Curry leaves (*Murraya koenigii* Spreng.)	Tree (p)	sapling (69)	100
Groundnut (*Arachis hypogaea* L.)	Nut	seed (10)	83
*Chowlai* (*Amaranthus gangeticus* L.)	GLV	seed (67)	86
Chakavat (*Chenopodium album* L.)	GLV	seed (43)	68
*Ambat Chuka* (*Rumex vesicarius* L.)	GLV	seed (52)	68
Winged bean (*Psophocarpus tetragonolobus* DC.) *tetragonolobus*)	Climber (p)	seed, sapling (67)	94
Tamarind (*Tamarindus indica* L.)	Tree (p)	seed, sapling (46)	99
Finger millet (*Eleusine coracana* Gaertn.)	Cereal	seed (23)	73
Custard Apple (*Annona squamosa* L.)	Tree	seed, sapling (32)	97
Field beans (*Dolichos lablab* L.)	Climber	seed, sapling (53)	94

(p) – Perennial

Trees of drumstick, *agathi* and curry leaves are perennial, fast growing and sturdy however, they would require care for the initial 1-2 years. Tamarind and custard apple are both sturdy trees with custard apple having the additional advantage of not being eaten by livestock. Tamarind is slow growing and fruits after 6-7 years though its leaves are available for consumption year round. Banana suckers can be used for propagation but the fruits are available only at one time in large quantities which is a disadvantage. Papaya and field beans are not valued by the community since they are widely available. Indian red spinach is a fast growing climber, gives a good yield and is a rich source of vitamins. It can grow on waste water and it requires minimal water and space. Winged beans also a climber can re-germinate automatically from tuber if planted successfully. All parts of winged beans like leaves, pods and tubers can be consumed making it highly advantageous. GLV's like *chakavat*, *ambat chukka* and *chowlai* are rich sources of vitamins. Garden cress, a rich source of iron, can be cultivated all year round with both leaves and seeds suitable for consumption. Other vegetables like turnip and cauliflower are also rich in vitamins and have the additional advantage that their leaves are also nutritious. Finger millet seeds, a rich source of vitamins and groundnut, having high caloric value and being able to withstand high temperatures in summer were the other plants distributed.

At the time of distribution; techniques of planting saplings and methods of vegetative propagation and seed harvesting were described along with their importance in the diet. Providing this information was critical as consumption habits are often governed by superstitions. Benefit of trees was also stressed upon to the HH members. Individual fencing of trees and climbers, to prevent destruction by livestock and ensure better survival, was promoted using locally available material. Nutrition based skills and knowledge of the community was built through recipe demonstrations for women and school children of the intervention plants. These were carried out once in every village during winter and 2 sessions if the village was large. *Subzi* and *paratha* of Indian red spinach and drumstick and *ladoo*s of garden cress were some of the recipes demonstrated. These sessions were also used as a means to educate the families on source and importance of micro-nutrients and a balanced diet. It was stressed that malnutrition was not just visible wasting and that micronutrient deficiency can be identified by signs of glossitis and stomatitis or weakness.

Education was a continuous process and was undertaken during follow-up visits and seasonal surveys. HHs were constantly motivated to increase their intake of GLVs, either fresh or dried, to approximately one cup per day. IEC booklets with pictorial representation of recipes of intervention plants, drying of leaves for consumption in summer and methods of propagation and water conservation were provided.

Since income generation did not necessarily translate into improved nutrition (Birdi, Joshi, Kotian, & Shah, 2014), the project chose not to have a livelihood component. Households were encouraged to harvest and distribute excess seeds to relatives and neighbours rather than setting up nurseries as a means of livelihood for a few.

### 3.3 Performance Indicators of the Intervention

The intervention saw many positive outcomes as well as challenges in terms of KG practices and food habits.

#### 3.3.1 KG Practices

All HHs now practiced KGs as opposed to 79% at baseline. Changes were seen in the season of cultivation, water source used as well as the location of the KG (Table 5).

**Table 5:** Comparison of KG location at baseline and end-line

Location of KG	Baseline – HH% (396 HHs)	Endline – HH% (362 HHs)
Specific area around the house	52	95
In the field	51	87
Along the walls/roof	45	96

KGs which were traditionally practiced during monsoon (98%) were now practiced by all HHs in monsoon and winter. Marked increase was seen during summer wherein 95% HHs maintained a KG as opposed to only 4% at baseline.

The water sources used for growing KG at baseline were rain water (83%), well water (26%), tap water (24%) and HH waste water (19%). Post intervention, a large number of HHs (63%) started using waste water for maintaining KGs in a specified area around the house or along the wall/roof of the house.

#### 3.3.2 Diet Diversity

Diet diversification was derived from the number of different types of food consumed in each category. First year of the study period (winter 2011 and summer 2012) was compared to the second year of the study period (winter 2012 and summer 2013) to see if there was any increase in diversity post intervention.

No change was seen in diversity for cereals and pulse in both seasons with majority of the HHs consuming 3 types of cereals and 2 types of pulses throughout the year. Change was seen in all other food categories (Table 6).

**Table 6:** Comparison of diet diversity at baseline and end-line

No. of different types of food items consumed	Winter 2011 (396 HHs)	Winter 2012 (259 HHs)	Summer 2012 (203 HHs)	Summer 2013 (362 HHs)
	GLVs (% of HHs)			
Not consumed	20*	0*	51^¶^	10^¶^
1-2	34	3	43	37
3-4	44	36	6^¶^	48^¶^
>4	2*	61*	0	5
	Roots/Tubers (% of HHs)			
Not consumed	3	0	7	0
1-2	83*	39	93	85
>2	15	61*	0^¶^	15^¶^
	Other vegetables (% of HHs)			
Not consumed	1	0	5	3
1-2	49	10	73	48
3-4	47*	85*	22^¶^	47^¶^
>4	3	5	0	3
	Fruits (% of HHs)			
Not consumed	13	1	24	5
Consumed	87*	99*	76^¶^	95^¶^
	Eggs (% of HHs)			
Not consumed	97	84	92	70
Consumed	3*	16*	8^¶^	30^¶^

* Values indicate a marked variation between two winters;

Values indicate a marked variation between two summers.

Winter: All the HHs during winter 2012 had consumed GLV as opposed to 80% HHs in winter 2011. Majority consumed 1-3 types of GLV at baseline (winter 2011) which increased to 4-5 types of GLVS in winter 2012. The number of HHs that consumed more than 2 varieties of root and more than 3 varieties of other vegetables also increased from 15% to 61% and 21% to 48% respectively. Fruits were consumed by almost all HHs during follow-up and an increase in variety was also observed. Egg consumption increased from 3% to 16%.

Summer: Number of HHs consuming GLVs increased from 49% (summer 2012) to 90% during the end of the study (summer 2013). Majority HHs had consumed 1-2 types of GLV (summer 2011) which increased to 3-4 types of GLVs during summer 2013. Most HHs consumed 2 varieties of roots but there were now about 15% HHs consuming >2 types of roots at the end of the study period. Number of HHs consuming > 3 varieties of other vegetables increased from 22% to 49% whereas HHs not consuming fruits reduced from 24% to 5%. Egg consumption increased from 8% to 30% towards the end of the study period (summer 2013)

#### 3.3.3 Quantity of Food Consumed in Each Category

Amount of food consumed in each category during the two winters and summers was also compared to the recommended dietary allowance per person/day (Table 7). Daily consumption per person was derived from weekly quantity purchased and divided by number of HH members above 5 years and an average obtained

**Table 7:** Seasonal comparison of daily average consumption per person per day under each food category

Food Category	Recommended dietary allowances for moderate activity^Ψ^ (gms)	Winter 2011 (gms)	Winter 2012 (gms)	Summer 2012 (gms)	Summer 2013 (gms)
Cereals	390	487	496.7	524.8	467.64
Pulses	82.5	88	58.9*	92.9	51.8*
GLV's	100	33.6	80.5	15.1	17.6
Roots	200	46.8	69.7	44.8	56.4
Other Veg	200	53	97.8	56	54.2

Ψ Obtained from second edition of National Institute of Nutrition, 2010.

* Pulse consumption reduced due to destruction of *tur* by unseasonal rainfall/pest.

Cereal consumption remained consistent throughout the study period since it was mainly obtained from the government public distribution system. Unseasonal rainfall and pest infestation resulted in destruction of pulse (*tur)* which contributed to decreased pulse consumption in the second year of the study. On comparing the consumption during baseline winter 2011 with winter 2012, a notable increase in consumption of GLVs, roots and other vegetables was seen. Summers did not see any notable increment in the quantity consumed.

During the 2 year study period, whilst diet diversification was achieved, the time was not enough to convince the community to increase the quantity consumed. Additionally, trees that were distributed required time to grow and therefore sufficient consumption of leaves was not possible. In fact some plants were over consumed due to which they did not survive and hence could not be further propagated.

#### 3.3.4 Childcare Practices

Qualitative intervention for childcare practices was restricted to inclusion of intervention plants in weaning. No change was observed in childcare practices before and after the study intervention. However, at least 16% HHs used the intervention plants for weaning and the most common ones were Indian red spinach (13 HHs), papaya (10 HHs) and Garden Cress (10 HHs). A few HHs also consumed drumstick (6 HHs), *ambat chukka* (4 HHs), banana (3 HHs), *agathi* (2 HHs), *chowlai* (2 HHs) and winged bean, tamarind, finger millet and groundnut (1 HH each).

### 3.4 Demand Generation

One of the most positive results of the study was an overwhelming response towards the KG intervention. The study saw a huge demand generation from HHs within the intervention villages and neighbouring villages. Since the plants introduced were either new to the area or rarely found, the community was keen to propagate them and the seeds were distributed to relatives and neighbours (19%). Some of the reasons for participation were consumption of vegetables (76%), to save money (10%) and to eat fresh vegetables (9%). Amongst the 411 newly added HHs, 81% practiced KG before the intervention. These KGs were mainly located in the field (84%) or in a specified area around the house (49%) and very rarely (3%) along the wall/roof or compound of the house. KGs were predominantly practiced in monsoon (98%) and winter (77%) and very rarely in the summer (5%). After participating in the study, HHs now practiced KGs mainly in a specified area around the house (80%) followed by the field (77%) and also along the wall/roof or compound of the house (65%). KGs were now practiced throughout the year in winter (100%), monsoon (98%) as well as summer (86%).

#### 3.4.1 Cohort vs. Demand Generated HHs

At the end of the study, certain parameters like survival, fencing and consumption of the intervention plants were noted for cohort HHs (362) as well as demand generated HHs (411). Demand generated HHs showed better fencing, survival and consumption for most of the intervention plants. Papaya, banana, custard apple, field bean, garden cress, cauliflower and *chakavat* were some of the intervention plants that showed better survival. Greater consumption was seen mainly for field bean, garden cress, groundnut and *chakavat* amongst the demand generated HHs.

Quantities averaged for male and female for comparison purpose. Moderate activity includes walking briskly (about 3½ miles per hour), climbing, gardening/yard work, dancing, walking short distances for fetching milk and vegetables, bicycling (less than 10 miles per hour), and weight training (a general light workout), *yoga* and *pranayama*, playing with children.

## 4. Discussion

Results from our study indicate that a substantial proportion of the caloric needs of the community are met through the Public Distribution System at subsidized rates (Birdi, Joshi, Kotian, & Shah, 2014). However, popularization and implementation of KG can aid in improving the diet diversity of the entire household and decrease nutrient deficiencies. Motivating HHs to sustain a KG once the organization leaves the study area could be one of the major drawbacks of a KG intervention. Keeping this in mind, our study focused on designing a perennial KG that included trees and climbers since motivation would only be required in the initial stages till the plants reach maturity. Our intervention also emphasised community participation by conducting cooking demonstrations which were followed by tasting sessions which aided in acceptance of the intervention plants. It was found that door to door interactions were most effective.

Water availability is a crucial consideration in any cultivation project. The Melghat region suffers from heavy rain with resultant flooding during the monsoon as well as extreme water shortage in summer. The study therefore addressed this by encouraging HHs to plant the creepers around the washing area and use waste water for watering the tree saplings. However, less success was achieved in protection of saplings during heavy rains. Some of the other reasons that could come in the way of a KG intervention were cited by HHs that did not participate in the project and these were either lack of time or no additional labour available for maintaining a KG.

Survival rates of the plants were not very good and a major reason for this other than flooding was destruction by livestock. All GLV's and other vegetables require adequate fencing and space for survival. Whilst the current project encouraged the use of local material for fencing, initial attempts were inadequate to prevent destruction. Excessive plucking of leaves by some HHs prevented further harvesting of seeds. An example for this was consumption of all greens of garden cress thus HHs were not able to harvest their seeds. In addition plucking of more than 10 % of the leaves affects the survival of trees (Rockwood, 1973). Other faulty practices like planting beneath big trees which blocked sunlight or planting two trees close together also contributed to lack of survival. All these highlight the lack of knowledge and the need for relevant education. This conclusion is supported by data published in India food security report that only 0.9% of farms have access to information diffused by *Krishi Vidyan Kendras* (Gahukar, 2011).

These factors also contributed to lack of significant increase in the consumption quantities of GLV's and other vegetables. The other major reason was that GLVs were consumed merely to increase palatability and change the taste rather than their nutritive value. It was observed that during winter a number of HHs perceived that they had a surplus of GLVs and therefore dried them. However the amount dried and consumed was inadequate. Thus the community requires education on drying sufficient quantities of excess harvest as well as unused parts of plants.

Most respondents preferred planting the intervention plants in their fields as they spent most time there. They perceived that water availability was better in their fields, however during summer the fields were often dry. It's during this period that the household waste water would still be available for KGs thus, emphasizing the importance of planting the saplings in the backyard.

Based on advantages and disadvantages of the 18 intervention plants, HHs perception and preferences we deduced the ideal intervention plants for Melghat (Table 8). The numbers of plants selected was kept to a minimum so as to not overwhelm the participants. In addition it was realized that it was necessary to estimate the amount of GLV produce that would be sufficient for each HH and based on this the number of saplings required for distribution of each variety per HH would need to be calculated. This would prevent excessive plucking and improve survival. Curry leaves, Garden cress were in high demand within the community and so were drumstick and Indian red spinach. Although the latter two were traditionally present in the community, they were not consumed due to lack of knowledge.

**Table 8:** Perennial KG design for Melghat

Type of plant	Part consumed	Nutrient content
Trees/shrubs		
Drumstick	leaf	pro-vitamin A, vitamin C, vitamin B_1_, B_2_, B_3_
Agathi	leaf	pro-vitamin A, vitamin C, calcium
Tamarind	pulp+ seed	iron, zinc, protein, vitamin B_1_, B_2_, B_3_
	leaf	vitamin B_1_, B_2_, B_3_
Curry leaf	leaf	pro-vitamin A, calcium, folate
Climbers		
Indian red spinach	leaf	pro-vitamin A, iron
Winged beans	leaf	pro-vitamin A, vitamin B_1_ & C
	seed	vitamin B_1_, iron, folate, protein, caloric value
Glv/nut		
Garden cress	leaf/seed	iron, seed also rich in vitamin B_3_, protein & caloric value
Groundnut (summer variety)	seed	vitamin B_3_, zinc, protein & caloric value

Besides the plants shortlisted above, guava trees could also be included as the fruit is nutritious and various parts of the tree have medicinal properties (Gutierrez, Mitchell, & Solis, 2008) including wide spectrum anti-diarrhoeal activity (Birdi et al., 2010; Birdi, Daswani, Brijesh, & Tetali, 2011). Correlation of diarrhoea and malnutrition is well established (Yunus, 2011).

Designing an intervention that looks separately at agriculture, health and nutrition is more viable than interventions that tackle complex problems (Burchi, Fanzo, & Frison, 2011). Previous projects conducted in Bangladesh (Nielsen, Roos, & Thilsted, 2003; Kumar & Quisumbing, 2010) which had income generation as their primary objective reported no significant effects of the food based intervention on energy, protein, vitamin A or iron intakes in children thus emphasizing that the livelihood component compromises good nutrition (Berti, Krasevec, & FitzGerald, 2004). A livelihood component in the present study was therefore not promoted and instead households were encouraged to harvest and distribute excess seeds to relatives and neighbours.

## 5. Conclusion

Targeting micronutrient deficiency requires altering a community's dietary habit which takes time and effort. Trying to increase the diet diversity by stressing on GLV's seems a more achievable method to eventually target micronutrient malnutrition. This study concludes that by providing communities with a well designed perennial KG that includes the right mix of plants for consumption along with health and nutrition knowledge can help in establishing a sustainable food and nutrition system which can ensure improved diet diversity, health and economic self-reliance.
